# Predictors of young people’s use of sexual and reproductive health services in Nigeria: a mixed-method approach

**DOI:** 10.1186/s12889-020-10022-x

**Published:** 2021-01-06

**Authors:** Amelia Ngozi Odo, Justina Ifeoma Ofuebe, Anthony Ifeanyi Anike, Efiong Sunday Samuel

**Affiliations:** Department of Human Kinetics and Health Education, University of Nigeria, Nsukka, Nigeria

**Keywords:** Sexual and reproductive health, Use, Predictors, Young people

## Abstract

**Background:**

Sexual and Reproductive health Services (SRHS) are essential for the prevention and control of SRH problems among young people and the achievement of sustainable development goal 3. These services may be available but certain factors interfere with their access and utilization by the young people. This study sought to determine factors that predict the utilization of SRHS among young people in Enugu State, Nigeria.

**Methods:**

The study adopted mixed-method research employing a cross-sectional research design. The population of the study comprised young people between the ages of 12 and 22 years. A multi-stage sampling procedure was used to select 1447 young people used for the study. A questionnaire, in-depth interview, and focus group discussion were used for data collection. Percentages, Chi-square, and logistic regression were used to analyse quantitative data, while qualitative data were thematically analysed using NVivo software.

**Results:**

Socio-demographic factors of gender, age, education, income, and living status (*p* = < .05) were significant predictors of utilization of SRHS. Psycho-cultural and health system factors (*p* = < .05) were also significant predictors of utilization of SRHS.

**Conclusion:**

The study concluded that some socio-demographic factors (of gender, age, level of education, income, and living status), psycho-cultural, and health system factors can be used to predict young people’s utilization of SRHS. These predictors could be addressed through home sex education, regular training of health care providers on youth-friendly services delivery, and policy reforms.

## Background

The world population is made up of slightly one-quarter of young people (10–24 years) [1]. In developing countries, this group constitutes 32% of the population [1] and is faced with several neglected health problems including sexual and reproductive health problems. In Nigeria, about 26.1% of the population is within 12-24 years [2]. Nineteen per cent of the young people (15–19) had begun childbearing [3] and are more likely to experience adverse pregnancy outcome than those who delayed childbearing. Moreover, the incidence and prevalence of sexually transmitted infections are high among young people aged 15–24 years [4, 5]. In 2018, 510,000 young people between the ages of 10 and 24 were newly infected with HIV, of whom 190,000 were adolescents between the ages of 10 and 19 years [6]. Young people are also at risk of other SRH problems such as unsafe abortion, early marriage, and sexual violence. They are exposed to these problems mainly due to their unhealthy sexual behaviours such as early sexual debut, multiple sexual partners, and unsafe sex [7]. Access to and utilization of Sexual and Reproductive Health Services (SRHS) are essential for the prevention of sexual and reproductive health (SRH) problems and diseases.

Though availability and accessibility of SRHS are still poor in developing countries [8, 9] including Nigeria, the little services that are available and accessible are underutilized by young people [10, 11]. Yet, there is an increase in the incidence and prevalence of SRH problems and diseases among them such as teenage pregnancy, unsafe abortion, sexually transmitted infections (STI), HIV, and AIDS in sub-Saharan Africa [12]. Young people are supposed to make effective use of these services because the services are meant to promote the sexual and reproductive health of every individual. The utilization of health services is measured based on health outcomes and the percentage of persons that use the services [13]. The significant impact of SRHS’ utilization can be observed in reproductive health outcomes such as pregnancy and birth, prenatal and neonatal mortality, maternal mortality, Sexually Transmitted Infections (STIs) and HIV and AIDS, and complications of unsafe abortion [14], since abortion is not legalized in Nigeria. The World Health Organization stated that nearly 20% of all global maternal deaths happen in Nigeria [15] with the risk higher among adolescent girls [16], suggesting that young people’s use of SRHS is low.

Many factors could determine the utilization of SRHS in Nigeria, despite efforts to make SRHS available at the primary healthcare facilities. These factors which are referred to as predictors in this study range from social, personal, psychological, and health system factors. The level of secrecy accorded to sexuality in some parts of Nigeria with its direct and indirect implications makes it difficult for sexually active young people to freely access and use SRHS, exposing a high percent of them to STIs [17]. It is, therefore, important to study the perceived predictors carefully to inform health professionals and policymakers which would enable them to understand the SRH challenges the young ones are facing and explore possible ways of addressing them. For this study, predictors were studied under the following subcategories: socio-demographic, psycho-cultural, and health system factors, to find out if they predicted young people’s utilization of SRHS. Socio-demographic factors include age, gender, level of education, religious affiliation, location, living status, marital status, economic status. Research has linked sociodemographic factors and young people’s utilization of health services [18, 19].

Moreover, one’s cultural and personal beliefs may influence the individual’s perception of accessing and using health services. Psycho-cultural refers to the interaction of psychological and cultural factors in the individual’s personality or the characteristics of a group [20]. Psycho-cultural factors in this study refer to those cultural beliefs or values that affect the psychology of the young one in seeking or using SRHS in Enugu State. Psycho-cultural factors included: the belief that discussing sexual issues is a taboo, fear of stigmatization or embarrassment based on cultural beliefs, fear of meeting their parents or people they know in the clinics, fear of being labeled a prostitute by community members, fear of not getting married later in life, fear of being barren, and other cultural beliefs regarding the use of SRHS by youths. For example, in some societies, most people assume that providing SRHS for the youth, like the provision of sexuality education and contraceptives, promotes promiscuity. These fears and burdens can limit adolescent’s use of SRHS and could result in stigmatizing youths that are bold enough to access and use available SRHS [21].

Furthermore, health system factors such as availability of quality reliable services, the proximity of the facility to users, cost of services, lack of privacy and confidentiality, long waiting time, using services with adults, and the attitude of service providers were assessed as predictors of young people’s utilization of SRHS. The nearer the facility to the users, the higher their level of access and utilization. Geographical access, therefore, influences service utilization [9, 22] The main objective of the present study was to determine if these factors predict the utilization of SRHS among young people in Enugu State. This has become necessary because such prediction studies are lacking in the State, while there are observed low utilization of health services among young people in Nigeria. We used young people and young ones interchangeably in this study.

## Methods

### Study area, design, and sampling techniques

Mixed-method research using cross-sectional design was conducted in Enugu State, Nigeria. One thousand, four hundred and forty-seven young ones (12–22 years) were randomly sampled through a multi-stage sampling procedure. In-depth Interviews (27) and focus group discussions (18) were conducted. The first phase of this study which focused on availability and accessibility of SRHS had been published [9]. Details of the study area, design, and sampling techniques are described in the study [9].

### Data collection procedure, processing, and analysis

A questionnaire (Additional File 1), in-depth interview guide (IDIG) (Additional File 2), and focus group discussion guide (FGDG) (Additional File 3) were used for data collection. The utilization of SRHS was measured using two sections of a questionnaire that was developed for a big project. Section A elicited information on the socio-demographic characteristics of the respondents while section B elicited information on the utilization of sexual and reproductive health services which include sexuality education, family planning services, safe motherhood services, post-abortion care, and prevention and treatment of STIs and HIV and AIDS. Section A of the questionnaire and part of the IDIG and FGDG which elicited information about the accessibility of SRHS have been published elsewhere (9). The Chi-square statistic and logistic regression were used to test the association at .05 level of significance. Other details about data collection, processing, and analysis are described in the published article (9).

Outcome variables (SRHS: sexuality education, family planning, safe motherhood, post-abortion care, and prevention and management of STIs and HIV/AIDS services) were measured dichotomously. Respondents were asked to indicate “Yes” if they have used or helped another young person use the services, otherwise “No”. Explanatory variables include Socio-demographic, psycho-cultural, and health system factors. The variables were categorically measured. Socio-demographic variables were gender, age, level of education, living status, location, and income status. The psycho-cultural variable was measured by the cumulative of responses to questions related to cultural beliefs and fear such as the belief that discussing sexual issues is a taboo, fear of stigmatization or embarrassment based on cultural beliefs, fear of meeting their parents or people they know in the clinics, fear of being labeled a prostitute by community members, fear of not getting married later in life, and fear of being barren. While health system variable was measured by the cumulative responses to questions relating to the health facility and service providers such as availability of quality reliable services, the proximity of the facility to users, cost of services, lack of privacy and confidentiality, long waiting time, using services with adults, and the attitude of service providers.

## Results

Table 1 shows the socio-demographic characteristics of the respondents. One thousand, four hundred and forty-seven (1447) young people between the ages of 12 and 22 years, with a mean age of 16.9 years responded to the questionnaire. More than half (57.1%) of the respondents were females while 42.9% were males. Slightly more than half (54.0%) had secondary education. The majority (56.7%) of the respondents lived in rural areas and most of them were living with their parents (62.3%). The majority had a monthly income of less than ₦5000.00 (1 USD = 199.3 NGN) [9].

**Table 1:** Socio-Demographic Characteristics of Young people that Responded to the Questionnaire on Utilization of SRHS (*n* = 1447)

Characteristics	%
**Gender**	
Male	42.9
Female	57.1
**Total**	**100.0**
**Age**	
12–16	48.2
17–22	51.8
**Total**	**100.0**
**Education**	
Primary	2.2
Secondary	54.0
Tertiary	42.0
None	1.8
**Total**	**100.0**
**Location**	
Urban	43.3
Rural	56.7
**Total**	**100.0**
**Living Status**	
With parents	62.3
Alone	20.4
With friends/husband	3.2
In school	14.1
**Total**	**100.0**
**Monthly Income**	
Below ₦1000.00 k	46.8
₦1000.00 k-₦4000.00 k	19.8
₦5000.00 k-₦10,000.00 k	16.0
₦11,000.00 k-₦20,000.00 k	8.8
Above ₦20,000.00 k	8.6
**Total**	**100.0**

Table 2 shows that the overall percentage total of 38.2 utilized SRHS. The table shows that more than half (56.6%) of the respondents reported using sexuality education services, 30.9% use family planning information and services, 36.5% use safe motherhood services, 23.6% use post-abortion care services, and 40.3% use services for the prevention and treatment of STIs and HIV and AIDS.

**Table 2:** Percentage Responses on the Utilization of SRHS for Youth in Enugu State (*n* = 1447)

Items Sexuality Education Services	%
Human biology	68.6
Puberty and menstrual hygiene education	72.0
Skill to overcome sexual desire	60.7
Healthy relationships	55.6
Dangers of pre-marital and unsafe sex	60.3
Counseling on reproductive health issues	48.6
Information on harmful cultural practices like female circumcision	46.0
Information on prevention of non-infectious conditions of reproductive health such as fistulas and cancers	41.2
**Cluster % Total**	**56.6**
**Family Planning Information and Services**	
Family planning information and counseling	42.2
Condoms	43.4
Oral pills	31.0
Injectable contraceptives	22.8
Intrauterine Contraceptive Devices (IUCDs)	22.0
Other contraceptives	23.8
**Cluster % Total**	**30.9**
**Safe Motherhood Services**	
Antenatal care	35.4
Skilled delivery	30.3
Postnatal care	33.4
Immunization	49.1
Infant feeding information	36.1
Growth monitoring	34.5
**Cluster % Total**	**36.5**
**Post Abortion Care Services**	
Emergency care during bleeding	25.8
Manual removal of retained product of conception	21.4
Information on prevention of unwanted pregnancy and abortion	36.8
Referral	23.6
**Cluster % Total**	**26.9**
**Services for Prevention and Treatment of STIs and HIV and AIDS**	
STIs and HIV and AIDS prevention information	56.5
Voluntary Counseling and Testing (VCT) for STIs and HIV	45.9
Antiretroviral therapy	22.9
Treatment of STIs	32.6
Condoms for prevention of STIs and HIV	43.8
**Cluster % Total**	**40.3**
**Overall % Total**	**38.2**

Table 3 shows that age (*p* = 0.00), level of education (*p* = 0.00), income (*p* = 0.03), psycho-cultural (*p* = 0.00), and health system (*p* = 0.00) are significantly associated with sexuality education utilization while gender (*p* = 0.05), age (*p* = 0.01), level of education (*p* = 0.05), location (*p* = 0.00), psycho-cultural (*p* = 0.00), and health system (*p* = 0.00) are significantly associated with the utilization of family planning. The table further shows that gender (*p* = 0.02), age (*p* = 0.03), level of education (*p* = 0.00), psycho-cultural (*p* = 0.00), and health system (*p* = 0.00) are significantly associated with safe motherhood services utilization. Age, level of education, location, income, psycho-cultural and health system (*p* = < 0.001) are significantly associated with the utilization of post-abortion care, while income (*p* = 0.02), psycho-cultural (p = 0.00), and health system (*p* = 0.00) are significantly associated with the utilization of services for the prevention and management of STI, HIV, and AIDS.

**Table 3:** Factors Associated with Utilization of SRHS (*n* = 1447)

Factors	Sexuality education			Family Planning			Safe motherhood			Post abortion care			Prevention and management of STIs, HIV and AIDS		
	%	X^2^	p	%	X^2^	P	%	X^2^	p	%	X^2^	p	%	X^2^	p
**Gender**															
Male	67.50	2.16	.14	36.10	3.73	.05	40.90	.02	.88	32.70	1.92	.16	42.20	3.05	.08
Female	71.10			31.20			41.30			29.30			37.70		
**Age**															
12–16	74.60	16.39	.00*	33.40	.01	.93	44.00	4.75	.03*	35.30	13.02	.00*	41.90	2.96	.09
17–22	64.80			33.20			38.40			26.50					
**Level of Education**															
Primary	40.60	15.50	.00*	40.60	8.04	.05*	21.9	26.68	.00*	21.90	16.01	.00*	28.10	5.97	.11
Secondary	69.50			32.40			42.10			34.40			40.50		
Tertiary	71.50			33.10			39.00			25.80			38.30		
None	57.70			57.70			84.60			46.20			57.70		
**Location**															
Urban	70.30	.34	.56	38.30	12.28	.00*	41.50	.06	.81	30.30	.10	.75	41.50	1.61	.20
Rural	68.90			29.50			40.90			31.10			38.20		
**Income**															
< 1000	72.50	11.03	.03*	36.50	6.90	.14	43.70	4.84	.30	36.30	18.95	.00*	42.70	11.46	.02*
1000–4000	70.60			32.20			40.20			26.20			39.50		
5000–10,000	68.10			28.90			35.80			24.60			34.90		
11,000–20,000	62.50			28.10			39.80			27.30			28.90		
> 20,000	60.50			32.30			40.30			25.80			42.70		
**Living status**															
With parents	68.50	5.84	.12	31.60	9.17	.03*	39.40	6.59	.09	30.60	2.65	.45	39.40	3.76	.29
Alone	69.50			40.70			47.10			33.90			43.40		
With friends	60.70			31.90			46.80			27.70			40.40		
In school hostel	76.00			30.40			38.70			27.50			34.80		
**Psycho-cultural**	73.20	9.89	.00*	38.90	21.45	.00*	47.40	24.93	.00*	37.70	34.34	.00*	48.20	47.31	.00*
**Health system**	72.30	11.99	.00*	35.90	10.37	.00*	42.90	4.33	.04*	32.70	5.72	.02*	42.60	12.48	.00*

*Significant

Table 4 shows that socio-demographic factors of gender; male (OR = 0.64; CI = 0.43–0.95), older age (OR = 0.23; CI = 0.14–0.38), and lower income (OR = 0.84; CI = 0.72–0.99) were significantly associated with lower odds of SRHS utilization, while tertiary education (OR = 2.93; CI = 1.93–4.46) and living in school (OR = 1.40; CI = 1.13–1.73), were significantly associated with higher odds of SRHS utilization. Similarly, psycho-cultural (OR = 2.40; CI = 1.50–3.84) and health system factors (OR = 7.47; CI = 4.66–12.01) were significantly associated with higher odds SRHS utilization.

**Table 4:** Predictors of Utilization of SRHS

Variables	Odds Ratio	***p***-value	(95% Conf. Interval)
Gender (male)	.64	0.03	.43–.95
Older ones (17–22)	.23	0.00	.14–.38
Tertiary Education	2.93	0.00	1.93–4.46
urban residence	1.39	0.10	.94–2.06
Lower monthly income (< 1000)	.84	0.03	.72–.99
Living in school	1.40	0.00	1.13–1.73
Psycho-cultural	2.40	0.00	1.50–3.84
Health system	7.48	0.00	4.66–12.01

### Qualitative data

Data generated through in-depth interviews using the IDIG reveal that only 9 (33.3%) out of 27 interviewees agreed that they have used other SRHS apart from sexuality education which 26 (96.1%) of them use in the schools, churches, and at homes. In the words of some interviewees:

*“I have never used any of these services”* (Enugu-North 002). *“I have only used sex education services” (Nkanu-West 002). “I don’t use them because I don’t think I need them”* (Udi 001). *“I did not use any of them … …… …*. , *though last year during youth week in my church, a health provider came and thought us about sex education”* (Udenu 002)*. “Yes, I used only sexuality education and services for prevention and management of STIs and HIV and AIDS”* (Ezeagu 002)*.* However, many of these interviewees did not want to reveal the specific services being used.

Few participants in the focus group discussions using the FGDG admitted using SRHS apart from sexuality education. Most males were of the view that SRHS is mainly for females except for sexuality education and services for prevention and management of STIs and HIV and AIDS. *“I have not used any of these services. They are only for females or married people”* (Udenu Male FGD-P1). *“It is true. P1 is correct”* (other Ps chorused)*. “Yes, at times we get some during youth week, seminars, and school”* (Udenu Female FGD-P7). *“I was tested for HIV last year”* (Nkanu-West Male FGD-P6). This implies that the majority of the participants use sexuality education and services for the prevention and management of STIs and HIV and AIDS only.

## Discussions

The utilization of sexual and reproductive health services among young people is essential to reduce the prevalence of SRH problems in developing countries [23, 24], which is posing a challenge to the actualization of SDG 3. Determining the factors that make young people use or not to use SRHS is very important in designing interventions to promote young people’s SRHS utilization. We utilized mixed method research because we have learned from experience that triangulating multiple methods of data collection is better than using a single method, especially when collecting sensitive data such as sexuality information. Our study found out that some socio-demographic, psycho-cultural, and health system factors could be used to predict the utilization of SRHS among young people.

The utilization of SRHS among young people was low. Sexuality education was the only SRHS utilized by slightly more than one-half of the respondents. The finding may be because these services, apart from sexuality education, were normally provided in the general health facilities which are not so comfortable for young people. Previous studies in other countries also reported [19, 25] low utilization of reproductive health services among young people. Similarly, qualitative data generated through in-depth interviews revealed that very few of the participants agreed that they had used other SRHS apart from sexuality education, which the majority of them used in the schools, churches, and homes. However, many of these interviewees did not want to reveal the services being used, which was not surprising to us because of the secrecy accorded to sexual issues generally and particularly in the study area. Few participants in IDI and FGD admitted using SRHS apart from sexuality education.

The utilization of sexuality education, safe motherhood, and post-abortion care services was associated with socio-demographic factors of age and level of education. There was an association between income level and sexuality education, post-abortion care, and services for the prevention and management of STIs, HIV, and AIDS while utilization of family planning services was associated with location, level of education, and living status. Younger respondents utilized sexuality education more than the older respondents. This could be because younger ones are still in school where sexuality education is taught, and the majority of the young people revealed in qualitative data that they used sexuality education services only in schools. The finding is consistent with [11] who reported that age is significantly associated with SRHS but the finding differs from Nisar and White [26], who reported no association between age and antenatal care utilization. Surprisingly, young people with no formal education used all the SRHS except sexuality education more than their counterparts with any formal education. Although some previous studies reported an association between level of education and utilization of SRHS [26–28], the common report has been that those with a higher level of education utilize the services more than those with no formal education.

All the SRHS were associated with psycho-cultural factors. Qualitative data revealed that young people in the study area believed that services under family planning are taboo for unmarried young people. The cultural belief is that family planning services are for married couples only. They also believed that SRHS will make youth promiscuous and barren later in life. These beliefs make some young people feel ashamed and afraid of using SRHS. This finding is consistent with previous research [29] which reported that cultural beliefs and practices affected utilization of maternal health services and one of the reasons young people do not use contraceptives includes feeling embarrassed or ashamed to use or purchase contraceptives [21]. Similarly, there is an association between all the SRHS and health systems factors. The finding could be because there is a lack of youth clinics or units which are expected to have specially trained service providers that provide youth-friendly SRHS. Information from the qualitative data revealed that most interviewees and FGD participants said that the pattern of service delivery like long waiting hours, lack of privacy, the attitude of health providers, and not being youth-friendly in services provision were the major health systems factors that influence their use of SRHS. The finding is consistent with Cheptum, et al. [29] who reported that lack of facilities, inadequate staffing, and negative staff attitude were associated with access and use of health services. Anusornteerakul, Khamanarong, Khamanarong, and Thinkhamrop [30] reported that the health service system is one of the important factors influencing the management of youth’s reproductive health services.

The finding showed that the male gender, older age, and lower-income were associated with lower odds of SRHS utilization while tertiary education and living in school, psycho-cultural and health system factors were associated with higher odds of SRHS utilization. These show that socio-demographic factors (gender, age, level of education, income, and living status), psycho-cultural, and health system factors could be used to predict young people’s utilization of SRHS. The findings agree with previous studies that reported some of these demographic factors as predictors of SRHS utilization [26, 31]. Previous studies also reported psychological and cultural factors as significant predictors of utilization of SRHS [29, 32]. The common reason young people do not use contraceptives included feeling embarrassed or ashamed to use or purchase condoms or any other contraceptives [33]. Additionally, the belief that discussing the sexual issue is a taboo prevents parents from rendering age-appropriate sex education at home, limiting young ones from getting basic information about sexuality early enough [11]. Health systems factors such as providers’ attitude, having a good and friendly relationship with the youth, keeping the client’s information confidential among others, determine youth’s access to reproductive health, and make youth reproductive health services successful. The proximity of health facilities, available services, and the good reputation of the providers were the main predictors for choosing health facilities [34, 35].

## Conclusion

The study concluded that some socio-demographic (of age, level of education, income, and living status), psycho-cultural and health system factors are predictors of young people’s utilization of SRHS. These predictors could be addressed through home sex education, regular training of health care providers on youth-friendly services delivery, and policy reforms.

### Limitations

The study utilized the cross-sectional design, therefore, cannot assume cause and effect association. The legal age of consent was a challenge because it was difficult to convince the parents of young people below the age of 18 years even when the young people were ready to participate. On the other hand, some young people declined their participation because their parents were to give consent. Nigeria is a multi-ethnic country and this study was conducted only in one state dominated by one ethnic, therefore, our findings may not be generalized to population or states dominated by other ethnic groups with different beliefs and perceptions about sexuality.

## Supplementary Information

**Additional file 1.** Questionnaire-SRHS. Predictors of access to and utilization of sexual and reproductive health services among youth in Enugu State, Nigeria. The questionnaire generated nominal data on the access and use of SRHS as well as predicting factors.

**Additional file 2.** In-depth Interview Guide (IDIG)-SRHS. Access to and Utilization on Sexual and Reproductive Health Services Among Youths in Enugu State. It generated qualitative data on individual’s experiences on access and use of SRHS

**Additional file 3.** Focus Group Discussion Guide (FGDG)-SRHS. Access to and Utilization on Sexual and Reproductive Health Services Among Youths in Enugu State. It generated qualitative data on small groups’ opinion on access and use of SRHS.

## Data Availability

The datasets generated and analyzed during the current study are available at Zenodo repository: https://zenodo.org/record/4294785#.X8J1wGhKg2w

## Abbreviations

FGD: Focus Group Discussion
HIV and AIDS: Human Immunodeficiency Virus and Acquired Immune Deficiency Syndrome IDI In-depth Interview
LGA: Local Government Area
SPSS: Statistical Package for the Social Sciences
SRH: Sexual and Reproductive Health
SRHS: Sexual and Reproductive Health Services
STIs: Sexually Transmitted Infections
WHO: World Health Organization
