# Supplementary material for: Predictors of young people’s use of sexual and reproductive health services in Nigeria: a mixed-method approach
Source: BMC Public Health. 2021 Jan 6;21:37. doi: 10.1186/s12889-020-10022-x (PMC7789390; doi:10.1186/s12889-020-10022-x)
Supplement: Supplementary file 1 — Additional file 1. Questionnaire-SRHS. Predictors of access to and utilization of sexual and reproductive health services among youth in Enugu State, Nigeria. The questionnaire generated nominal data on the access and use of SRHS as well as predicting factors. [file 12889_2020_10022_MOESM1_ESM.docx]

**Predictors of Access and Utilization of Sexual and Reproductive Health Services (SRHS) Questionnaire (PAUSReQ)**

Department of Health and Physical Education,

University of Nigeria, Nsukka.

Dear Respondent,

This questionnaire is designed to identify access and utilization of Sexual and Reproductive Health Services (SRHS) and also factors that predict youths’ access and utilization of SRHS in Enugu State. This will help improve access and utilization of SRHS among the youth. You are kindly required to respond to each item of the questionnaire as honestly and frankly as possible. There is no right and wrong answers. Your responses will be treated confidentially and none of them will be held against you. Please do not write your name in any part of the form.

Thanks for your co-operation.

Yours Sincerely,

**Odo, Amelia. N.**

**Section A: Personal Data**

**Instruction:** Please place a tick **(√)** against the option that best applies to your response in each item.

**1**. What is your gender? Male Female

**2**. What is your age? 12-16 17-22

**3**. What is your level of education?

a. Primary Education

b. Secondary Education

c. Tertiary Education

d. None Formal Education

**4**. What is your Religious affiliation?

a. Christianity

b. Islam

c. African Traditional Religion

**5.** Where do you reside (Location)?

a. Urban

b. Rural

**6**. Who are you living with?

a. Parents

b. Alone

c. Friends

d. Live in school

**7.** What is your Marital Status?

a. Married

b. Single

c. Divorced

d. Separated

**8.** If married, how many children do you have? (Parity)

a. None

b. One-three

c. Four-six

d. Seven and above

**9**. What is your estimated Monthly income/pocket money?

a. Below ₦1,000.00k

b. ₦1,000.00k - ₦4,000.00k

b. ₦5,000.00k - ₦10,000.00k

c. ₦11,000.00k - ₦20,000.00k

d. Above ₦20,000.00k

**Section B: Accessibility to Sexual and Reproductive Health Services (SRHS).**

**Instruction:** Please indicate how the SRHS below are accessible to you by placing a tick **(√)** against the option that best applies to your response in each item.

| **S/N** | **Services** | **Geographical Access** |  | **Financial Access (cost of transport and services)** | |
| --- | --- | --- | --- | --- | --- |
|  | **The following are youths’ sexual and reproductive health services.** | **Accessible (can walk in within 30mins or not more than 1 mile away)** | **Not accessible (can’t walk in within 30mins or more than 1 mile away)** | **Affordable** | **Not Affordable** |
| 10 | Sexuality education services such as education on: |  |  |  |  |
|  | 1. human biology |  |  |  |  |
|  | 1. puberty |  |  |  |  |
|  | 1. menstrual hygiene |  |  |  |  |
|  | 1. skills to overcome sexual desires |  |  |  |  |
|  | 1. healthy relationship |  |  |  |  |
|  | 1. dangers of premarital sex |  |  |  |  |
| **S/N** | **Services** | **Geographical Access** | | **Financial Access (cost of transport and services)** | |
|  |  | **Accessible (can walk in within 30mins or not more than 1 mile away)** | **Not accessible (can’t walk in within 30mins or more than 1 mile away)** | **Affordable** | **Not Affordable** |
| 11 | Family planning information and services such as: |  |  |  |  |
|  | 1. condoms |  |  |  |  |
|  | 1. oral pills |  |  |  |  |
|  | 1. Injectables |  |  |  |  |
|  | 1. intrauterine contraception, |  |  |  |  |
| 12 | Safe motherhood services such as |  |  |  |  |
|  | 1. Antenatal |  |  |  |  |
|  | 1. Delivery |  |  |  |  |
|  | 1. Post natal |  |  |  |  |
|  | 1. Immunization |  |  |  |  |
|  | 1. Infant feeding information |  |  |  |  |
| 13 | Post Abortion (or miscarriage) Care services such as: |  |  |  |  |
|  | 1. emergency care during bleeding |  |  |  |  |
|  | 1. manual removal of retained product of conception |  |  |  |  |
|  | 1. information on prevention of unwanted pregnancy |  |  |  |  |
|  | 1. information on prevention of abortion |  |  |  |  |
| 14 | Prevention and treatment of STIs and HIV and AIDS information and services such as |  |  |  |  |
|  | 1. voluntary counseling and testing |  |  |  |  |
|  | 1. provision of antiretroviral therapy(ART) |  |  |  |  |
|  | 1. treatment of STIs |  |  |  |  |
|  | 1. supply of condoms |  |  |  |  |
|  | 1. prevention of mother-to-child transmission of HIV and other STIs |  |  |  |  |

**Section C: Utilization of Sexual and Reproductive Health Services (SRHS).**

**Instruction:** Please place a tick **(√)** against the option that best applies to your response in each item.

| **S/N** | **Items** | **Yes** | **No** |
| --- | --- | --- | --- |
|  | **Sexuality Education Services:** Have you received from accessible health facility any of these services within the last one year? Information or Education on: |  |  |
| 15 | Human biology e.g. structure and functions of reproductive system for youth? |  |  |
| 16 | Puberty and menstrual hygiene practices e.g. characteristics of puberty, washing of underwear and other personal hygiene practices? |  |  |
| 17 | Skills to overcome sexual desire e.g. decision making and refusal skills? |  |  |
| 18 | Healthy relationships e.g. dating or courtship? |  |  |
| 19 | Dangers of pre-marital and unsafe sex for youth e.g. unwanted pregnancy, Sexually Transmitted Infections and HIV etc? |  |  |
| 20 | Counseling on reproductive health issues like reproductive rights and policy for youth? |  |  |
| 21 | Information on harmful cultural practices like female circumcision? |  |  |
| 22 | Information on prevention of non-infectious conditions of reproductive health such as fistula and cancers? |  |  |
|  | **Family Planning Information and Services:** Have you **received** or **used** any of these services from the accessible health facility within the last one year? |  |  |
| 23 | Family planning information and counseling |  |  |
| 24 | Condoms |  |  |
| 25 | Oral pills |  |  |
| 26 | Injectable hormones (e.g. depo) |  |  |
| 27 | Intrauterine contraceptive devices (e.g. copper T or loop) |  |  |
| 28 | Other contraceptives |  |  |
|  | **Safe Motherhood Services:** Have you **received or used** any of these services from accessible health facility within the last one year? |  |  |
| 29 | Antenatal care (ANC) |  |  |
| 30 | Skilled delivery(delivery conducted by a trained health worker) |  |  |
| 31 | Post natal care |  |  |
| 32 | Immunization |  |  |
| 33 | Infant feeding especially exclusive breast feeding information |  |  |
| 34 | Growth monitoring |  |  |
|  | **Post Abortion (or miscarriage) Care (PAC) Services:** Have you received or used any of these services from accessible health facility within the last one year? |  |  |
| 35 | Emergency care during bleeding |  |  |
| 36 | Manual removal of retained product of conception |  |  |
| 37 | Information on prevention of unwanted pregnancy and abortion |  |  |
| 38 | Referral |  |  |
|  | **Prevention and treatment of STIs and HIV and AIDS:** Have you received or used any of these services from accessible health facility within the last one year? | **Yes** | **No** |
| 39 | STIs and HIV and AIDS prevention information |  |  |
| 40 | Voluntary Counseling and Testing (VCT) for STIs and HIV |  |  |
| 41 | Antiretroviral therapy |  |  |
| 42 | Treatment of STIs |  |  |
| 43 | Condoms for prevention of STIs and HIV |  |  |

**Psycho-Cultural and Health System Factors:** Below are factors which could influence your access to or utilization of the above SRHS? Please indicate by placing a tick (√) against the option that best applies to your response in each item.

| **S/N** | **Items** | **Yes** | **No** |
| --- | --- | --- | --- |
|  | **Psycho-Cultural Factors** |  |  |
| 44 | Belief that discussing sexual issues is a taboo |  |  |
| 45 | Fear of stigmatization based on cultural beliefs regarding the use of SRHS by youth |  |  |
| 46 | Fear of meeting my parents or people that I know |  |  |
| 47 | Fear of being labeled a prostitute by the community members |  |  |
| 48 | Fear of being barren later in life |  |  |
|  | **Health System Factors** |  |  |
| 49 | Proximity (nearness) of the facility |  |  |
| 50 | Cost of services |  |  |
| 51 | Lack of desired privacy and confidentiality |  |  |
| 52 | Long waiting time |  |  |
| 53 | Using services with older ones |  |  |
| 54 | Attitude of service providers |  |  |

**Section D: Suggested Ways of Improving Youths’ Access to and Utilization of SRHS**

**Instruction:** Please indicate by placing a tick (√) against the option that best applies to your response in each item.

| **S/N** | **Items** | **Yes** | **No** |
| --- | --- | --- | --- |
|  | Youth’s access to and use of SRHS can be improved by: |  |  |
| 55 | Providing SRHS for youth only in separate youth clinics |  |  |
| 56 | Youth clinics should be close to where young people always gather like schools, churches, amusement parks, community, markets and sports facility. |  |  |
| 57 | Training of providers on characteristics of youth-friendly services |  |  |
| 58 | Making SRHS youth-attractive and friendly |  |  |
| 59 | Youth’s SRHS should be free or as low-cost as possible |  |  |
| 60 | Youths should be involved in services design and delivery |  |  |
| 61 | Minimizing waiting time |  |  |
| 62 | Permitting walk-in appointment (that is attending to client whenever the client comes to the clinic) |  |  |
| 63 | Offering as many services as possible in a location |  |  |
| 64 | Improving workers attitude towards young ones access and use of SRHS |  |  |
| 65 | Convenient opening hours |  |  |
| 66 | Continuity of service delivery |  |  |
| 67 | Correction of some cultural misconceptions about youth access and use of SRHS |  |  |
| 68 | Awareness campaign on the need to start sexuality education from home by parents |  |  |
| 69 | Comprehensive sexuality education in schools |  |  |
| 70 | Maintaining Privacy and respecting individual’s personality |  |  |
