# Supplementary material for: Predictors of young people’s use of sexual and reproductive health services in Nigeria: a mixed-method approach
Source: BMC Public Health. 2021 Jan 6;21:37. doi: 10.1186/s12889-020-10022-x (PMC7789390; doi:10.1186/s12889-020-10022-x)
Supplement: Supplementary file 2 — Additional file 2. In-depth Interview Guide (IDIG)-SRHS. Access to and Utilization on Sexual and Reproductive Health Services Among Youths in Enugu State. It generated qualitative data on individual’s experiences on access and use of SRHS [file 12889_2020_10022_MOESM2_ESM.docx]

**In-Depth Interview Guide (IDIG)**

**Access and Utilization on Sexual and Reproductive Health Services Among Youths in Enugu State**

**Target Group: Youths (12-22 years)**

Introduction

Thank you for giving me your precious time today to meet with you. My name is …………… from University of Nigeria, Nsukka. I would like to ask you questions regarding issues on availability, accessibility and utilization of sexual and reproductive health services (SRHS) among youths, as well as those factors that make you to use or not to use SRHS and ways you think youths’ access and utilization of SRHS could be improved on. I will be asking you questions just to understand what you know, feel or your experiences towards seeking and using SRHS.

I promise to keep all responses confidential. Though, I will take note and also tape record your responses, only the research team will have access to listening to the recorded conversations. Recording is just because I do not want to miss any of your comments, contributions, responses and questions. Please yourcooperation and patience are highly needed. The interview will not last more than 40 minutes and so will not take much of your precious time. However, your participation is completely voluntary.

**Are you willing to participate? Yes ( ) No ( ) or do you have any question, clarifications, comment or suggestions before we continue.**

**……………………………………….. ………………………………**

**Interviewee Date**

**……………………………………….. …………………………….**

**Parent (if interviewee is under 18 years) Date**

| **S/N** | **Questions** | **Probe Guide** |
| --- | --- | --- |
|  | Create rapport by greeting, good entry behaviour, collect socio-demographic variables of interest. |  |
| 1 | Do you think that the following sexual and reproductive health services are being provided for youths?   1. Sexuality education services 2. Family planning information and services 3. Safe motherhood services like antenatal, skilled delivery, post natal, etc 4. Post abortion care services 5. Prevention and management of STIs and HIV and AIDS | - What and what are available for youths? - Where can you get these services? - Who provides them? - What information do you have? - Any restriction to using them? |
| **2** | Do you have access to these SRHS services?   1. Sexuality education services 2. Family planning information and services 3. Safe motherhood services like antenatal, skilled delivery, post natal, etc 4. Post abortion care services 5. Prevention and management of STIs and HIV and AIDS | - Can you walk up to the health facility that provides these services within 30 minutes or not? - Are you able to pay for the transport fare and cost of the services? |
| **3** | Do you use these SRHS services?   1. Sexuality education services 2. Family planning information and services 3. Safe motherhood services like antenatal, skilled delivery, post natal, etc 4. Post abortion care services 5. Prevention and management of STIs and HIV and AIDS | - When did you use any of them last? - Where did you use it? At home, school, friends’ house? - Who provided the services (trained or not)? - How were these services provided for youths? Hostile or friendly? |
| **4** | Which of the following do you think can influence youths’ access to SRHS   1. Fear of meeting my parents or relatives 2. Fear of being labeled a prostitute 3. Fear of being barren later in life 4. Proximity of health facility that provide the service 5. Pattern of service delivery like waiting hours, privacy, and youth-friendly services 6. Attitude of service providers | - How and why? - What have you heard about the use of SRHS like family planning, post abortion care and others by youths in your culture? - What belief do you have about youth SRHS in this community? - Restrictions from family and community - Community and family taboos. - What are those factors inherent in the health facility that contribute to either use or not of SRHS among youths? |
| **5** | How do you think that youths’ access and utilization of these services could be improved on? | - What can be done by youths, society, health facility, service providers, and government? |
| **6** | When was the last time you used any of these services? What was your experience with using them? | - How did you know about the service(s) - Where did you get the service? - How did you feel? - What encouraged you? Or - What discouraged you? |
