# Supplementary material for: Predictors of young people’s use of sexual and reproductive health services in Nigeria: a mixed-method approach
Source: BMC Public Health. 2021 Jan 6;21:37. doi: 10.1186/s12889-020-10022-x (PMC7789390; doi:10.1186/s12889-020-10022-x)
Supplement: Supplementary file 3 — Additional file 3. Focus Group Discussion Guide (FGDG)-SRHS. Access to and Utilization on Sexual and Reproductive Health Services Among Youths in Enugu State. It generated qualitative data on small groups’ opinion on access and use of SRHS. [file 12889_2020_10022_MOESM3_ESM.docx]

**Focus Group Discussion Guide (FGDG)**

**Access and Utilization on Sexual and Reproductive Health Services Among Youths in Enugu State**

**Target Group: Youths (12-22 years)**

**Introduction**

Thank you all for giving us your precious time today to meet with us. We are research assistants from University of Nigeria, Nsukka. We would like to discuss with you the issues on availability, accessibility and utilization of sexual and reproductive health services (SRHS) as they relate to you, the young ones, as well as those factors that make you to use or not to use SRHS. We will be asking you questions just to understand what you know, feel or your experiences towards seeking and using SRHS.

We promise to keep all responses confidential. Though, we will take note and also tape record your responses, only the research team will have access to listening to the recorded conversations. Recording is just because we do not want to miss any of your comments, contributions, responses and questions. Please this discussion will not last more than 40 minutes and so will not take much of your precious time. However, your participation is completely voluntary.

**Can we continue with the discussion? Yes ( ) No ( ) or do you have question, clarifications, comment or suggestions before we continue.**

| 1 | Do you think that the following sexual and reproductive health services are being provided for youths?   1. Sexuality education services 2. Family planning information and services 3. Safe motherhood services like antenatal, skilled delivery, post natal, etc 4. Post abortion care services 5. Prevention and management of STIs and HIV and AIDS | Probe for What? How, by who and under what condition? |
| --- | --- | --- |
| **2** | Do you have access to these SRHS services? | Geographical and financial? |
| **3** | Do you have access to these SRHS services? | How and when? |
| **4** | What are those factors that make you to access these services? | What and why? |
| **5** | What are those factors that make you to use or not to use these services? | What and why? |
| **6** | What do you think could be done to improve youths’ access to and utilization of SRHS could be improved? | How? Who? |
